# The Platelet–Adipose Tissue Axis in Metabolic Disease: From Platelet Reprogramming to Tissue Remodeling

**DOI:** 10.3390/cells15201863

**Published:** 2026-10-13

**Authors:** Rosa Simone, Cosimo Damiano Perrone, Valentina Lemmo, Chiara Lonobile, Silvia Stella Barbieri

**Affiliations:** 1 Unit of Neuro-Cardiovascular Axis, Centro Cardiologico Monzino IRCCS, 20138 Milan, Italy; rosa.simone@cardiologicomonzino.it (R.S.); cosimo.perrone@cardiologicomonzino.it (C.D.P.); valentina.lemmo@cardiologicomonzino.it (V.L.); chiara.lonobile@cardiologicomonzino.it (C.L.); 2 Department of Biology and Biotechnology “L. Spallanzani”, Università degli Studi di Pavia, Via Bassi 21, 27100 Pavia, Italy; 3 Department of Theoretical and Applied Sciences, eCampus University, 22060 Novedrate, Italy

**Keywords:** platelet, adipose tissue, cardio-metabolic disease

## Abstract

Obesity-induced adipose tissue remodeling results from the interplay of metabolic stress, chronic inflammation, extracellular matrix remodeling, and vascular dysfunction. Beyond their established role in thrombosis, platelets have emerged as immunometabolic effectors at the interface between vascular and metabolic responses. This review examines mechanistic, translational, and clinical evidence supporting a platelet–adipose tissue axis and its relevance to obesity-associated cardiometabolic disease. In obesity and related metabolic disorders, platelets display a hyperreactive phenotype characterized by alterations in glycolysis, mitochondrial function, oxidative stress, and secretory activity, together with changes in extracellular vesicle biogenesis and cargo. Through direct cellular interactions and soluble or vesicle-mediated signaling, platelets may influence leukocyte recruitment, macrophage phenotype, fibroblast activation, extracellular matrix remodeling, and endothelial or adipocyte metabolic pathways relevant to adipose tissue dysfunction. However, the extent of direct platelet-dependent effects within adipose tissue is poorly known. We propose that these mechanisms may participate in a feed-forward thromboinflammatory circuit in which metabolic stress promotes platelet reprogramming, while platelet-derived signals may in turn amplify endothelial dysfunction, inflammation, fibrosis, and impaired adipose tissue plasticity. Although direct causal evidence in humans remains limited, current data support a biologically coherent framework in which platelets may act as amplifiers of adipose tissue dysfunction rather than established primary drivers. Overall, this platelet–adipose tissue axis may provide a framework for investigating novel molecular pathways relevant to cardiometabolic risk stratification and therapeutic strategies.

## 1. Introduction

Adipose tissue is a highly dynamic immunometabolic organ that integrates endocrine, vascular, neural, and immune signals to maintain systemic metabolic homeostasis [1,2,3]. Its ability to expand and remodel in response to nutrient excess depends on coordinated adaptations involving adipocytes, stromal and immune cells, extracellular matrix, and tissue vasculature [1,3,4]. Under physiological conditions, this multicellular network preserves metabolic homeostasis, tissue expandability, and vascular integrity.

In obesity, however, this adaptive capacity is progressively impaired. Adipocyte hypertrophy, inadequate vascularization, extracellular matrix expansion, and local hypoxia promote cellular stress, immune cell recruitment, endothelial activation, and fibrotic remodeling [1,5,6,7,8]. The resulting loss of adipose tissue plasticity contributes to insulin resistance, ectopic lipid deposition, and increased cardiovascular risk [1,4,7,9,10,11]. Although adipocytes, immune cells, and stromal populations are the principal drivers of adipose tissue remodeling, increasing evidence suggests that circulating and vascular cells may also contribute to these processes. Among them, platelets have emerged as key regulators at the intersection of thrombosis, inflammation, and metabolism [1,12]. Beyond their canonical role in hemostasis, platelets modulate leukocyte trafficking, endothelial activation, inflammatory signaling, and intercellular communication through direct cell–cell interactions, soluble mediators, and extracellular vesicles [12,13,14,15].

Obesity and type 2 diabetes are associated not only with enhanced platelet reactivity but also with alterations in platelet metabolism, including changes in bioenergetics, mitochondrial function, redox homeostasis, calcium signaling, and granule secretion [8,16,17,18,19,20,21,22]. These alterations may have functional consequences beyond increased platelet reactivity. Whereas platelet hyperreactivity reflects an enhanced response to activating stimuli, metabolic reprogramming may also modify platelet signaling and secretory output. Activated and metabolically altered platelets release bioactive molecules including CD40L, PF4/CXCL4, CCL5, serotonin, growth factors, and extracellular vesicles carrying regulatory miRNAs [13,15,23,24,25,26,27]. Through direct cellular interactions and soluble or vesicle-mediated signaling, platelets may therefore influence inflammatory, vascular, and metabolic processes relevant to adipose tissue remodeling [1,4,13,16,28]. Whether these platelet alterations merely reflect systemic metabolic and vascular stress or actively contribute to adipose tissue dysfunction remains unclear.

Current evidence supports multiple mechanistic links between platelet activation and pathways implicated in adipose tissue dysfunction; however, direct tissue-level and causal evidence, particularly in humans, remains limited. Platelets may therefore act as amplifiers of the reciprocal interactions among vascular dysfunction, chronic inflammation, and metabolic stress, rather than as primary drivers of adipose tissue remodeling.

In this review, we examine the mechanistic, translational, and clinical evidence supporting the platelet–adipose tissue axis in obesity-associated cardiometabolic disease. We first summarize the key features of adipose tissue remodeling and then discuss alterations in platelet phenotype and metabolic programming in metabolic disease. We next integrate evidence for platelet-mediated immune amplification, structural remodeling, and metabolic regulation, distinguishing established mechanisms from emerging or putative interactions. Finally, we evaluate the strength and limitations of current experimental and human evidence and discuss their implications for biomarker development, cardiometabolic risk stratification, and targeted therapeutic intervention. Particular attention is given to whether metabolic reprogramming modifies platelet effector function in ways that may contribute to adipose tissue dysfunction.

## 2. Adipose Tissue Remodeling

Adipose tissue remodeling is an adaptive process that enables adipose depots to accommodate changes in nutrient availability while preserving metabolic homeostasis. In obesity, persistent energy overload progressively exceeds this adaptive capacity, resulting in alterations in adipocyte biology, immune composition, extracellular matrix architecture, and vascular function [8,10,29,30,31]. Here, we focus on the remodeling features most relevant to vascular exposure and potential communication with platelets. The transition from adaptive expansion to maladaptive remodeling promotes chronic low-grade inflammation, fibrosis, and impaired metabolic flexibility, contributing to systemic metabolic dysfunction and increased cardiometabolic risk.

### 2.1. Adipocyte Hypertrophy Versus Hyperplasia

Adipose tissue expansion occurs through adipocyte hypertrophy (increase in cell size) and hyperplasia (increase in cell number), with important consequences for metabolic health [9,32,33]. Hyperplastic expansion generally supports lipid storage while preserving tissue function, whereas excessive hypertrophy is associated with mitochondrial and endoplasmic reticulum stress, impaired insulin signaling, altered lipid handling, and the release of pro-inflammatory adipokines and danger-associated molecular patterns (DAMPs) [10,29,32,33,34,35,36]. Adipocyte enlargement also increases the oxygen diffusion distance and contributes to local hypoxia [7,37]. Thus, the balance between hypertrophic and hyperplastic expansion influences whether adipose tissue adaptation progresses towards metabolic dysfunction [32,36,38,39].

### 2.2. Immune Cell Recruitment and Inflammatory Reprogramming

Obesity profoundly changes the immune landscape of adipose tissue [10,11]. Resident macrophages increase in number and shift toward a pro-inflammatory phenotype, often accumulating around necrotic or dying adipocytes in crown-like structures [11,40,41], while additional innate and adaptive immune populations contribute to the inflammatory microenvironment [42,43,44].

Chemokines, including CCL2 (MCP-1) and members of the CXCL family, promote the recruitment of circulating monocytes and other leukocytes, while local cytokine production sustains immune activation [45,46]. These interactions reinforce local inflammation, adipocyte dysfunction, and insulin resistance [10,11], and actively contribute to tissue remodeling [8,45].

This inflammatory microenvironment provides an interface between local adipose tissue dysfunction and circulating immune and vascular components [10,11,12,16,43,47,48,49].

### 2.3. Extracellular Matrix Remodeling and Fibrosis

Extracellular matrix (ECM) remodeling is a major determinant of adipose tissue plasticity in obesity [4,8,50]. Chronic nutrient excess promotes excessive deposition of collagens, fibronectin, and other matrix components, increasing tissue stiffness and limiting adipose tissue expansion [4,50,51]. Fibroblast activation and profibrotic signaling, particularly through transforming growth factor-β (TGF-β), contribute to progressive ECM accumulation [4,9,50]. Increased ECM stiffness and impaired tissue expansion further exacerbate cellular stress and metabolic dysfunction [4,8,52,53].

### 2.4. Angiogenesis and Hypoxia

Adipose tissue expansion requires coordinated vascular adaptation [7]. Inadequate angiogenic responses during rapid weight gain lead to insufficient perfusion, promoting regional hypoxia, endothelial dysfunction, and activation of hypoxia-responsive pathways, particularly HIF-1α signaling [6,7,54]. These responses favor inflammatory and profibrotic gene expression.

The adipose microvasculature constitutes the principal interface between the circulation and the tissue compartment [55]. Endothelial cells within adipose depots regulate leukocyte trafficking and maintain vascular integrity [28,56,57]. In obesity, endothelial activation and increased permeability facilitate immune cell recruitment, while inflammatory and hypoxic signals further impair vascular function [6,45]. The resulting interplay between hypoxia and vascular dysfunction promotes maladaptive tissue remodeling [6,7,28,56,57].

### 2.5. Depot-Specific Remodeling: Visceral, Subcutaneous, and Perivascular Adipose Tissue

Adipose tissue remodeling in obesity is highly depot-specific [47,58,59]. Subcutaneous and visceral adipose tissues differ in cellular composition, vascular architecture, expandability, and inflammatory propensity, with important consequences for systemic metabolic risk [39,59,60].

Subcutaneous adipose tissue generally exhibits a greater capacity for hyperplastic expansion and lipid storage [32,39,59], whereas visceral adipose tissue is more prone to hypertrophy, hypoxia, immune-cell infiltration, and inflammatory activation [8,43,58,59,60].

Perivascular adipose tissue (PVAT) represents a specialized adipose depot at the interface between metabolic and vascular homeostasis because of its close anatomical relationship with the vessel wall [61,62]. Under physiological conditions, PVAT contributes to vascular homeostasis through vasoactive and anti-inflammatory signals [62]. However, in obesity, PVAT undergoes phenotypic switching characterized by increased oxidative stress, inflammatory activation, immune cell accumulation, and loss of its normal anti-contractile properties [61,63], thereby contributing to endothelial dysfunction and vascular stiffness.

Differences in vascularization, endothelial phenotype, immune composition, and inflammatory activity are likely to influence tissue exposure and responsiveness to blood-borne signals. Visceral adipose tissue and, in particular, PVAT may therefore represent sites where metabolic, inflammatory, and vascular signals converge. These features suggest that the impact of circulating factors, including platelet-derived mediators, may vary according to adipose tissue localization.

## 3. Platelet Activation and Metabolic Reprogramming in Metabolic Disease

Obesity and related metabolic disorders are consistently associated with alterations in platelet phenotype and function [21,22,64]. Beyond their established contribution to thrombotic risk, platelets in metabolic disease exhibit enhanced activation together with changes in intracellular metabolic and redox pathways that may influence their inflammatory and immunomodulatory functions [12,16,21]. Hyperglycemia, dyslipidemia, insulin resistance, oxidative stress, endothelial dysfunction, and chronic low-grade inflammation collectively create a systemic environment that promotes platelet hyperreactivity and may alter platelet metabolism [22,64,65,66]. These metabolic changes may affect not only platelet responsiveness but also the signals released following activation.

### 3.1. Platelet Hyperreactivity in Obesity and Type 2 Diabetes

Individuals with obesity and type 2 diabetes display increased platelet aggregation, enhanced expression of activation markers such as P-selectin, and increased integrin αIIbβ3 activation [21,22,64,67]. Circulating platelets also show increased sensitivity to classical agonists, including thrombin and collagen, and enhanced adhesion to activated endothelium [64,65].

Multiple metabolic abnormalities converge on intracellular pathways that lower the threshold for platelet activation. Hyperglycemia promotes non-enzymatic glycation of platelet proteins and increases intracellular calcium mobilization [68,69]. Experimental evidence further indicates that hyperglycemia increases mitochondrial superoxide production in human platelets, leading to inhibition of protein tyrosine phosphatase activity and enhanced phosphorylation of Syk during collagen-dependent signaling [70]. Altered Ca^2+^ handling provides an additional mechanism contributing to platelet hyperreactivity in diabetes. In human diabetic platelets, changes in the direction and activity of Na^+^/Ca^2+^ exchange favor Ca^2+^ influx and contribute to enhanced intracellular Ca^2+^ responses, while prolonged exposure of normal platelets to high glucose can reproduce similar alterations [71].

Insulin resistance further weakens physiological inhibitory signaling in platelets [21,72,73]. Consistent with this mechanism, the antiaggregatory effect of insulin is impaired in platelets from individuals with obesity and from obese patients with type 2 diabetes [74]. Oxidized low-density lipoproteins provide an additional link between dyslipidemia and platelet hyperreactivity. Engagement of platelet CD36 by oxidized low-density lipoproteins activates Src- and PKC-dependent signaling, promotes NOX2-derived ROS generation, and impairs inhibitory cGMP/PKG signaling, thereby enhancing platelet activation [75]. More recently, increased N-linked glycosylation of platelet CD36 has been demonstrated in patients with type 2 diabetes and linked to enhanced oxidized low-density lipoprotein-mediated Lyn–JNK signaling [76].

Mitochondrial dysfunction and increased reactive oxygen species (ROS) production can further lower the activation threshold and enhance platelet signaling [65]. Together, dysregulated Ca^2+^ homeostasis, impaired insulin-dependent inhibition, and enhanced oxidative signaling sustain a pro-thromboinflammatory platelet phenotype that favors interactions with activated endothelium and circulating leukocytes [21,64,67].

Recent human evidence further indicates that obesity and type 2 diabetes exert additive effects on platelet hyperreactivity and activation, accompanied by increased platelet oxidative stress and reduced nitric oxide bioavailability [67].

### 3.2. Metabolic Reprogramming of Platelets

Platelets are metabolically active cells that rely on coordinated glycolytic and mitochondrial pathways to sustain basal function and meet the rapid energetic demands associated with activation [19,77,78,79]. Metabolic flux analysis in human platelets has shown that activation markedly increases glucose consumption and redistributes central carbon metabolism, underscoring the close coupling between platelet bioenergetics and functional responses [80].

In obesity and related metabolic disorders, systemic changes in glucose and lipid availability, insulin signaling, and oxidative stress may alter this metabolic balance.

Changes in glucose utilization, mitochondrial function, oxidative phosphorylation, redox homeostasis, and intracellular calcium signaling have been associated with abnormal platelet responsiveness [16,19,21,77]. In this context, mitochondrial bioenergetics and NADPH oxidase (NOX)-mediated ROS generation represent important regulators of platelet activation thresholds and pro-inflammatory signaling [81,82]. In patients with type 2 diabetes and vascular complications, combined bioenergetic and metabolomic analyses have identified impaired mitochondrial respiration, altered NAD^+^/NADH balance, increased glycolytic flux, and enhanced mitochondrial oxidant generation, providing evidence that metabolic disease can be associated with an altered platelet bioenergetic phenotype [83].

These metabolic changes are not equivalent to platelet activation itself. Hyperreactivity describes an increased functional response to activating stimuli, whereas metabolic reprogramming reflects alterations in the intracellular pathways that support platelet signaling and effector functions. Emerging data suggest that platelet metabolic alterations may have effects beyond classical hemostasis, raising the possibility that platelets participate in pathways relevant to peripheral lipid homeostasis and systemic insulin sensitivity [84]. Changes in glycolytic flux, mitochondrial activity, and ROS generation may therefore influence both activation thresholds and processes involved in granule secretion and extracellular vesicle formation [19,77].

Importantly, metabolic influences on platelet phenotype may begin upstream of circulating platelets. Megakaryocyte maturation requires extensive remodeling of lipid metabolism, including fatty-acid uptake and de novo lipid synthesis [85]. Particularly relevant to obesity, experimental evidence indicates that increased bone-marrow adiposity can influence megakaryopoiesis. In a high-fat diet-induced obesity model, transfer of adipocyte-derived fatty acids to megakaryocytes promotes megakaryocyte maturation and proplatelet formation through a CD36-dependent mechanism [86]. These findings provide evidence that obesity-associated changes in the adipose environment can influence platelet production upstream at the megakaryocyte level. Whether similar metabolic cues contribute to the altered platelet phenotype observed in human obesity, however, remains to be established.

Collectively, these findings indicate that platelet hyperreactivity and metabolic reprogramming in obesity and type 2 diabetes reflect the convergence of multiple metabolic and signaling alterations rather than a single disease-specific pathway. Key mechanisms and their experimental context are summarized in Table 1. Whether these alterations simply enhance platelet responsiveness or also qualitatively modify platelet effector and secretory functions remains unresolved.

### 3.3. Altered Platelet Secretory Phenotype

Activated platelets release a broad repertoire of bioactive molecules stored in α-granules and dense granules or generated following activation, extending platelet responses beyond hemostasis [12,15]. These signals include chemokines such as PF4/CXCL4 and CCL5, inflammatory mediators including CD40L and IL-1β, growth factors such as TGF-β and PDGF, and dense-granule constituents including platelet-stored serotonin [15,87].

In obesity and related metabolic disorders, enhanced platelet activation is accompanied by increased degranulation and altered circulating levels of platelet-derived mediators [21,88]. These molecules can regulate endothelial, immune, and stromal cell functions and may therefore provide a potential link between systemic platelet activation and responses in peripheral tissues [12,13].

Metabolic alterations may also influence the composition of platelet-derived signals. However, whether they generate a distinct, disease-associated secretory profile independently of changes in platelet reactivity remains unclear. Changes in mitochondrial function, redox balance, and intracellular signaling may influence granule release and mediator availability, but whether such changes produce a reproducible secretory phenotype relevant to adipose tissue remains to be established.

### 3.4. Platelet-Derived Extracellular Vesicles

Extracellular vesicles (EVs) released by activated platelets represent an additional mechanism of intercellular communication [89,90,91]. Platelets are the predominant source of circulating EVs, and platelet-derived EVs carry proteins, lipids, adhesion molecules, and regulatory RNAs whose composition may reflect the functional and metabolic state of the parent platelet [11,89,92]. Indeed, circulating small EVs (sEVs), predominantly originating from activated platelets and vascular cells, are emerging as crucial inter-organ messengers capable of carrying disease-specific bio-cargo under thromboinflammatory conditions [93,94,95].

Obesity and related metabolic disorders have been associated with quantitative and qualitative alterations in platelet-derived EVs. Circulating platelet-derived EVs have been reported to be increased in individuals with severe obesity compared with lean controls [96,97,98,99]. Analysis of EVs released from thrombin-activated platelets of obese and non-obese individuals showed no difference in their number but revealed increased size heterogeneity and substantial differences in their proteomic composition, suggesting that obesity may influence the phenotype and molecular cargo of platelet-derived vesicles rather than simply their abundance [96].

Among their regulatory cargo, miR-223 and miR-126 have been implicated in inflammatory signaling, endothelial function, and metabolic homeostasis [25,26,100]. A direct mechanism of platelet EV-mediated molecular transfer has been demonstrated for miR-223: microvesicles released from thrombin-activated human platelets contain functional Ago2-miR-223 complexes, are internalized by endothelial cells, and regulate endogenous gene expression in recipient cells [25,26,100]. EV-mediated transfer may therefore provide a mechanism through which platelet signaling occurs beyond sites of direct cell–cell contact.

Their ability to circulate beyond sites of platelet activation and interact with vascular cells makes platelet-derived EVs candidate mediators of communication between circulating platelets and metabolic tissues. However, whether obesity alters the molecular cargo of platelet-derived EVs in a reproducible manner and whether these vesicles cross the adipose microvascular barrier and deliver their cargo to specific cell populations within adipose tissue remain unclear.

**Figure 1:**
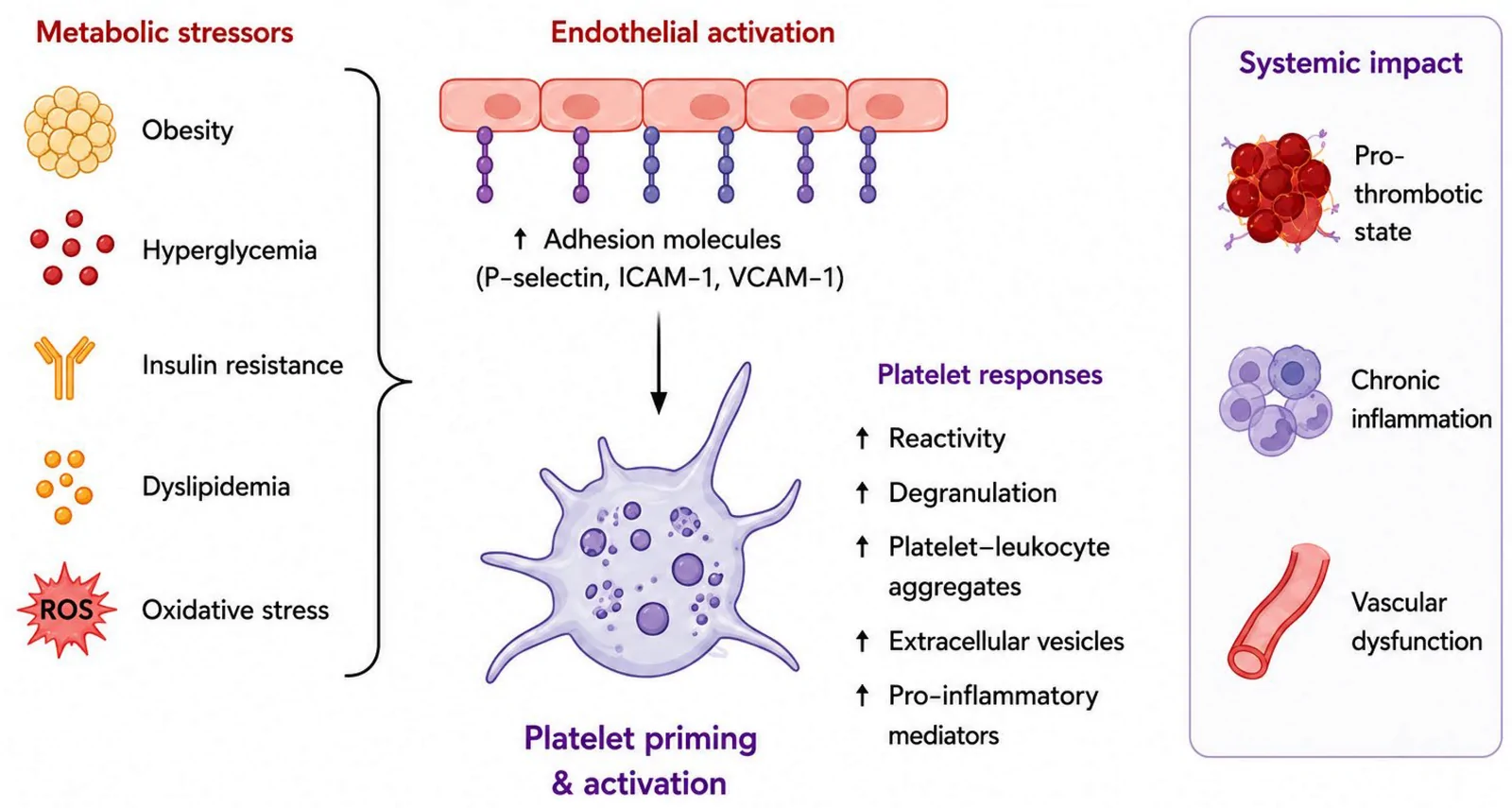
Platelet Activation in Metabolic Disease. Chronic metabolic stressors—including obesity, hyperglycemia, insulin resistance, dyslipidemia, and oxidative stress—induce endothelial activation and upregulation of vascular adhesion molecules (P-selectin, ICAM-1, VCAM-1). This environment drives platelet priming and hyperreactivity, characterized by enhanced degranulation, increased formation of platelet–leukocyte aggregates, elevated release of small extracellular vesicles (sEVs), and a potentially altered secretory phenotype. ICAM-1, Intercellular Adhesion Molecule 1; ROS, Reactive Oxygen Species; VCAM-1, Vascular Cell Adhesion Molecule 1. The figure was generated using ChatGPT-5.6 sol Enterprise (OpenAI; DALL·E).

Together, these metabolic, oxidative, and vascular alterations converge to promote platelet priming and activation in metabolic disease (Figure 1).

## 4. Platelets in Immune, Structural, and Metabolic Remodeling

The functional diversity of platelets enables them to link vascular activation with immune, stromal, and metabolic signaling [12,16]. In metabolic disease, persistent platelet activation and metabolic alterations may increase the availability or modify the profile of platelet-derived signals that act on cells within adipose tissue [21]. These effects can be organized into three interconnected levels: immune activation, structural remodeling, and metabolic regulation. Evidence supporting a platelet contribution, however, differs considerably among these processes.

### 4.1. Immune Amplification

The adipose microvasculature provides a potential interface where activated platelets may influence leukocytes and the endothelium; however, direct access of intact platelets to the adipose interstitium and the extent to which platelet-derived products cross the endothelial barrier have not been established. Nevertheless, experimental evidence supports local platelet interactions within the adipose microvasculature. Using intravital fluorescence microscopy, Nishimura et al. demonstrated enhanced leukocyte–endothelial cell–platelet interactions in the microcirculation of visceral adipose tissue in obese mice, together with increased platelet adhesion to the vascular wall, local platelet P-selectin expression, and monocyte–platelet conjugate formation [101]. These findings provide direct evidence of platelet activation at the adipose vascular interface, but do not establish extravasation of intact platelets into the adipose interstitium. Under metabolic stress, endothelial activation increases the expression of adhesion molecules, including P-selectin, ICAM-1, and VCAM-1, facilitating adhesion and platelet-leukocyte interactions. Activated platelets bind circulating monocytes through P-selectin–PSGL-1 interactions and additional integrin-dependent mechanisms, promoting platelet–monocyte aggregate formation, leukocyte activation, and transendothelial migration [15,17]. The platelet-monocyte axis has recently been shown to amplify thromboinflammatory responses and promote endothelial IL-1R signaling in obesity, supporting the link between obesity and vascular damage [102].

Beyond adhesion-dependent mechanisms, platelet-derived mediators can modulate inflammatory signaling in endothelial cells and monocytes. Engagement of CD40 on endothelial cells and monocytes by platelet-derived CD40L activates NF-κB signaling and induces the expression of inflammatory cytokines and chemokines, including TNF-α, IL-6, and CCL2 [13,103]. Platelet-derived PF4/CXCL4 and CCL5 can also promote monocyte recruitment and influence monocyte differentiation [13,24,104].

Within lipid-rich adipose microenvironments, platelet-derived signals may interact with fatty acid-induced lysosomal stress and the metabolic pathways associated with metabolically activated macrophages (MMe) [24,105]. PF4 signaling has been implicated in monocyte differentiation and suppression of alternative macrophage polarization [24,104], and could therefore favor the persistence of an inflammatory macrophage phenotype. Direct evidence demonstrating that platelet-derived PF4 drives macrophage reprogramming specifically within obese adipose tissue, however, remains limited.

Platelet-derived extracellular vesicles may also contribute to immune regulation by transferring regulatory microRNAs and signaling molecules to recipient cells [89]. EV-associated miRNAs, including miR-223, can modulate inflammatory gene expression and metabolic pathways [25,26]. Their contribution to macrophage reprogramming within human adipose tissue, however, has not been established.

Together, platelet adhesion, soluble mediators, and EVs provide several plausible routes through which activated platelets may influence leukocyte recruitment and inflammatory signaling associated with adipose tissue dysfunction. However, the extent to which platelet-derived signals cross the endothelial barrier and act within the adipose interstitium remains unresolved. Thus, current evidence more strongly supports the adipose vascular interface as a site of platelet–adipose communication than direct interaction of intact platelets with cells within the adipose interstitium.

### 4.2. Structural Remodeling: Fibrosis and Vascular Adaptation

Fibrosis and vascular maladaptation are major determinants of impaired adipose tissue plasticity in obesity [4,8,50]. Platelets contain and release several mediators with established profibrotic activity, including TGF-β and platelet-derived growth factor (PDGF) [9,87]. Platelets represent an important circulating reservoir of TGF-β1, which is stored in platelet α-granules and released upon activation [106]. Similarly, platelet α-granules contain PDGF, which can promote fibroblast proliferation and chemotaxis [107,108]. TGF-β promotes myofibroblast differentiation and collagen deposition, whereas PDGF contributes to stromal-cell proliferation and matrix remodeling [9,27,109]. In adipose tissue, PDGFRα signaling in perivascular stromal progenitors has also been shown to promote profibrotic conversion and extracellular matrix deposition [110]. Release of these mediators from activated platelets could contribute to profibrotic signaling, but platelet-specific effects within adipose tissue have not been established in vivo.

However, the specific contribution of platelet-derived TGF-β or PDGF cannot be separated from that of the same mediators produced by immune, endothelial, and stromal cells. Thus, although platelet-derived TGF-β1 and PDGF have established biological effects relevant to fibrosis, a direct platelet-derived TGF-β1/PDGF–adipose fibrosis pathway has not been demonstrated. The extent to which platelets contribute to the local profibrotic milieu in obese adipose tissue is therefore unknown.

Platelets also store and release regulators of vascular remodeling, including vascular endothelial growth factor (VEGF) and other angiogenic mediators, such as brain-derived neurotrophic factor (BDNF) [9,15,111]. Given that platelets represent the primary circulating reservoir of BDNF, evaluating its peripheral dynamics requires careful methodological standardization, particularly in obese subjects where metabolic stress alters neurotrophin release and vascular homeostasis [112]. Because adequate vascular expansion is required to maintain oxygen delivery during adipose tissue growth, platelet-derived angiogenic signals may contribute to vascular adaptation. Conversely, persistent platelet activation in obesity could alter the balance of pro- and anti-angiogenic signals and contribute to abnormal microvascular remodeling. Direct evidence for these effects in adipose tissue remains limited.

Platelet-derived signals may therefore influence vascular components of adipose tissue remodeling, although their tissue-specific contribution in vivo has not been quantified. A similar pathogenic role for hyperreactive platelets has been documented in metabolic steatotic liver disease, where platelet-derived mediators accelerate immune cell infiltration and fibrotic tissue remodeling [113]. These findings support a broader thromboinflammatory role of platelets in metabolic disease but should not be assumed to demonstrate a direct platelet-adipose mechanism.

### 4.3. Platelet-Derived Signals and Adipose Cell Metabolism

Evidence that platelet-derived signals directly alter adipocyte metabolism is more limited than that supporting platelet interactions with immune or vascular cells. Nevertheless, serotonin and platelet-derived EVs provide two potential routes of metabolic regulation.

Platelet-stored serotonin is a potential mediator. Platelets take up, store, and release serotonin generated primarily in extra-platelet tissue, thereby contributing to its peripheral bioavailability [114]. Serotonergic signaling through specific receptors, including 5-HT2A and 5-HT2B, in adipocytes modulates lipid metabolism and thermogenic regulation [23,115]. Increased peripheral serotonin signaling can promote lipid accumulation and suppress thermogenic programs, including pathways associated with UCP1 expression and mitochondrial oxidative metabolism [23,115]. Altered platelet activation may change the release of stored serotonin and thereby influence peripheral serotonergic exposure, but whether this platelet contribution independently alters adipose tissue metabolism has not been demonstrated in vivo.

Platelet-derived extracellular vesicles provide another potential route of platelet-adipose cross-talk. Their regulatory microRNAs, including miR-223, can influence inflammatory and insulin-responsive pathways in recipient cells [25,26]. Although uptake of platelet-derived EVs by several vascular and immune cell populations has been demonstrated, their direct effects on adipocytes and adipose stromal populations remain less well characterized. It is also unclear whether obesity-associated changes in EV cargo alter insulin signaling, lipid handling, or mitochondrial function within adipose tissue.

These observations suggest that changes in platelet metabolism could influence adipose tissue indirectly by modifying platelet secretion and EV cargo. Direct evidence linking this altered output to adipocytes or stromal-cell dysfunction, however, is still limited.

**Figure 2:**
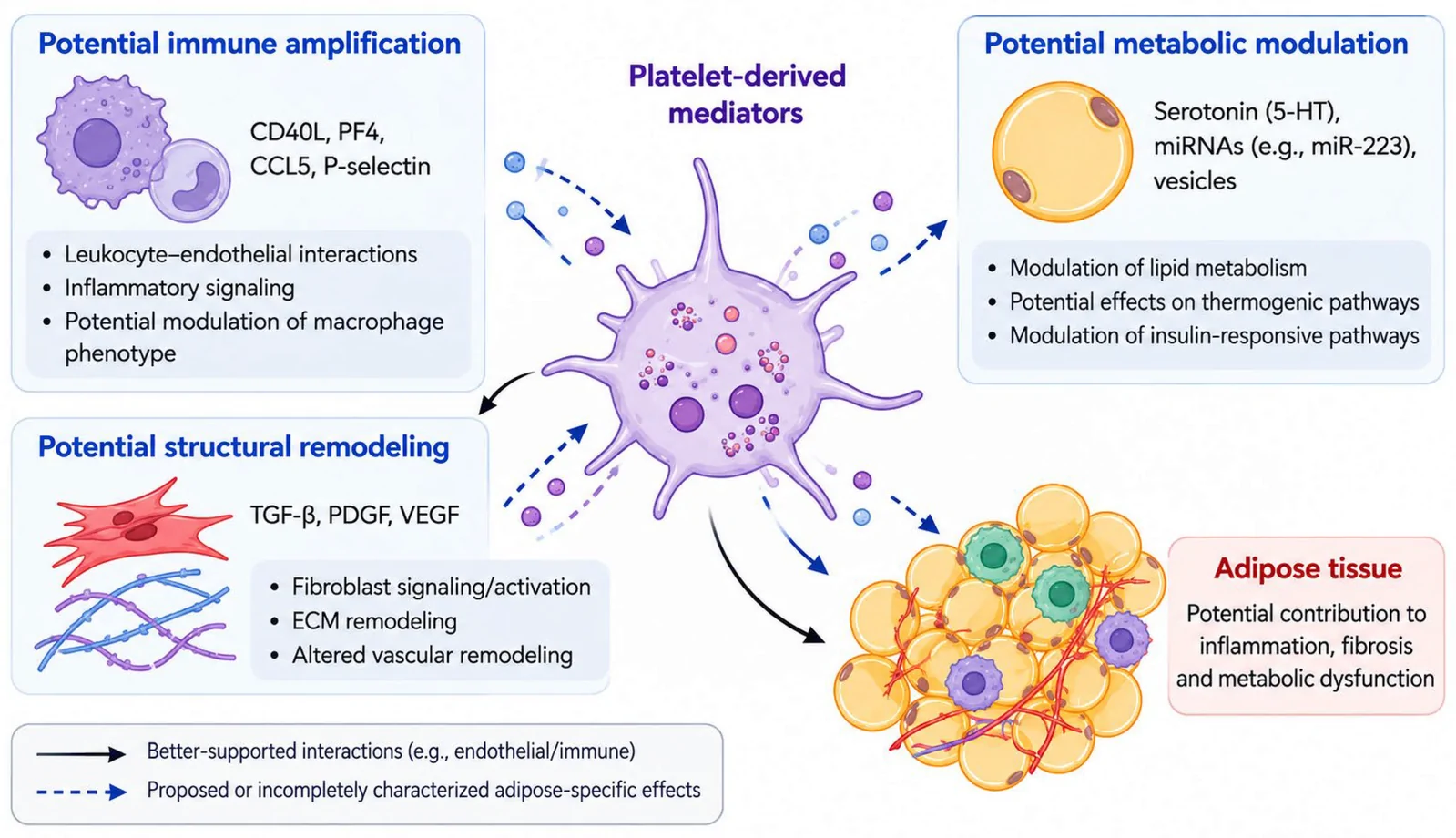
Platelet–Adipose Tissue Interactions. Activated platelets may influence cellular pathways relevant to the adipose tissue microenvironment through three interconnected pathways: (i) Immune amplification, via surface molecules such as P-selectin and soluble mediators including CD40L, PF4/CXCL4, and CCL5, which can enhance leukocyte-endothelial interaction and inflammatory signaling; (ii) structural remodeling, via profibrotic and angiogenic growth factors such as TGF-β, PDGF, and VEGF, whose effects on stromal and vascular cells are established in other contexts, although the specific contribution of platelet-derived mediators within adipose tissue remains poorly defined; (iii) potential metabolic regulation via platelet-stored serotonin (5-HT) and vesicle-mediated transfer of regulatory microRNAs (e.g., miR-223), for which direct platelet-dependent effects on adipose tissue remain to be established. 5-HT, 5-Hydroxytryptamine (Serotonin); CCL5, C-C Motif Chemokine Ligand 5; CD40L, CD40 Ligand; ECM, Extracellular Matrix; miR-223, MicroRNA-223; PDGF, Platelet-Derived Growth Factor; PF4, Platelet Factor 4; TGF-β, Transforming Growth Factor beta; VEGF, Vascular Endothelial Growth Factor. The figure was generated using ChatGPT-5.6 sol Enterprise (OpenAI; DALL·E).

Together, these observations identify potential immune, structural, and metabolic pathways through which platelet-derived signals may influence adipose tissue remodeling, although the strength of evidence differs substantially across these mechanisms and direct tissue-level causality remains limited (Figure 2).

### 4.4. The Platelet–Adipose Tissue Axis

**Figure 3:**
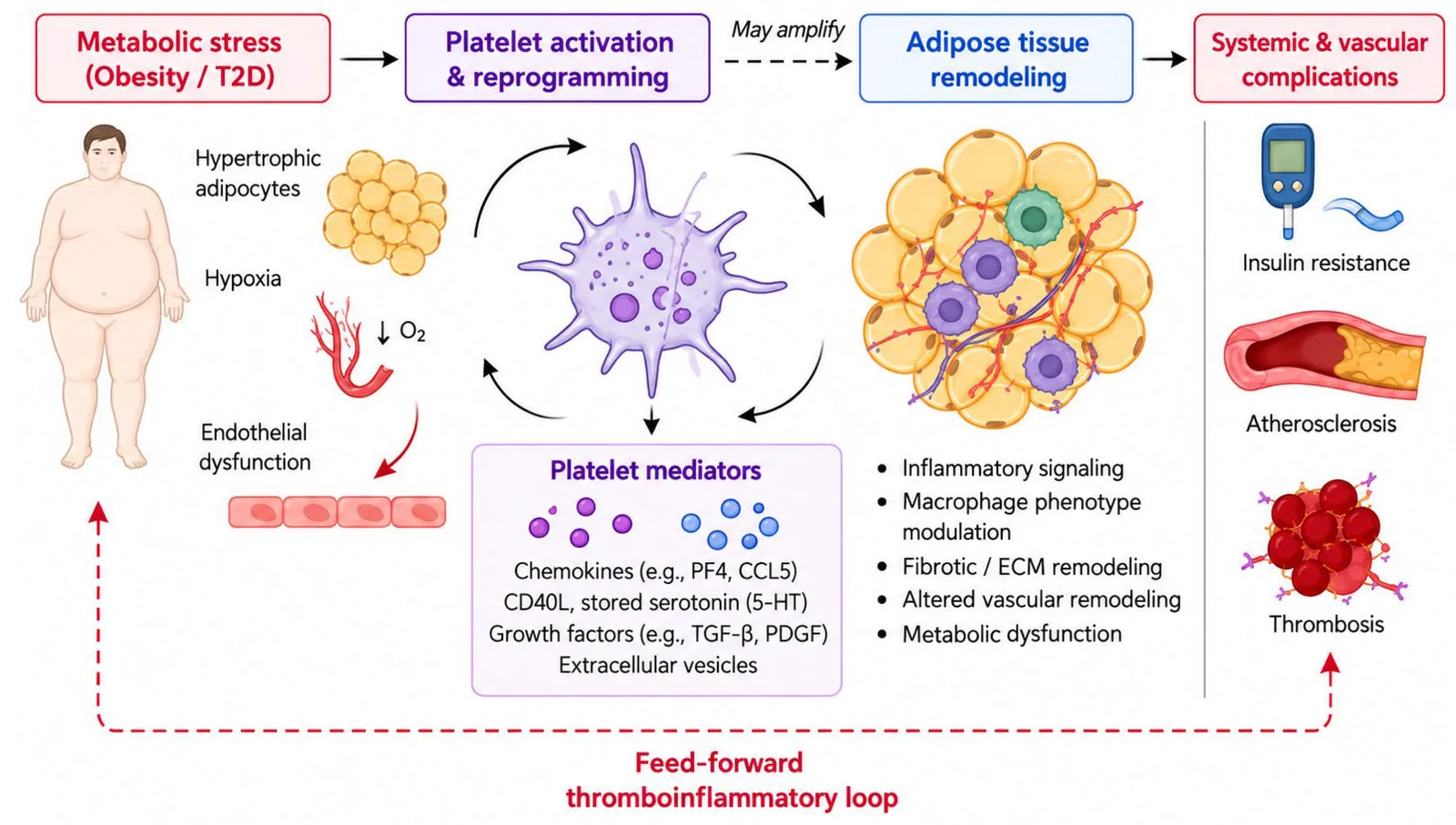
Potential Integrative Model: The Platelet–Adipose Axis. Systemic metabolic stress and adipose tissue dysfunction (hypertrophic adipocytes, tissue hypoxia, and endothelial activation) may form a self-sustaining, feed-forward thromboinflammatory loop. Platelet-derived mediators may amplify selected inflammatory, vascular, stromal, and metabolic pathways associated with adipose tissue dysfunction. However, direct platelet-dependent effects within adipose tissue and their contribution to downstream systemic complications remain to be established. O_2_, Oxygen; T2D, Type 2 Diabetes; TGF-β, Transforming Growth Factor beta. The figure was generated using ChatGPT-5.6 sol Enterprise (OpenAI; DALL·E).

Collectively, the immune, structural, and metabolic functions of platelets may contribute to a thromboinflammatory circuit that links vascular activation to maladaptive adipose tissue remodeling (Figure 3).

In obesity, adipocyte hypertrophy, hypoxia, and inflammatory signaling promote endothelial activation and leukocyte recruitment [5,7,17]. At the same time, systemic metabolic abnormalities promote platelet hyperreactivity and metabolic alterations that may modify platelet interactions with leukocytes and the endothelium as well as their secretory and vascular output [19,21,77].

Activated platelets may enhance local inflammation through P-selectin-dependent leukocyte tethering and CD40L-mediated NF-κB activation, promoting monocyte recruitment and inflammatory macrophage polarization within adipose depots [13,15,24]. Platelet-derived PF4 may further influence macrophage differentiation toward pro-inflammatory phenotypes that intersect with metabolic pathways characteristic of metabolically activated macrophages (MMe) [24,105]. The resulting inflammatory milieu can promote fibroblast activation, extracellular matrix deposition, and progressive tissue stiffening through profibrotic mediators, including TGF-β [9,27]. The extent to which glycolytic and 1-carbon metabolic pathways influence granule release or extracellular vesicle cargo selection in metabolically stressed platelets remains an open question [79,116]. The main candidate molecular mediators, cellular targets, and proposed biological effects are summarized in Table 2.

In parallel, platelet-derived serotonin and extracellular vesicles may modulate adipocyte metabolic programs and inflammatory or insulin-responsive pathways [23,25,26,115]. Progressive inflammation and fibrosis impair adipose tissue plasticity and exacerbate hypoxia and lipotoxic stress, further sustaining endothelial dysfunction and a systemic environment that favors platelet activation [6,7,64].

Platelet dysfunction may, in turn, amplify selected inflammatory, vascular, stromal, and metabolic pathways within susceptible adipose depots. This reciprocal model is particularly relevant to visceral and perivascular adipose tissue, where vascular, inflammatory, and metabolic signals converge.

Importantly, the individual components of this framework are supported by different levels of evidence. Platelet hyperreactivity and platelet–leukocyte interactions in metabolic disease are well established, whereas direct causal evidence for platelet-driven fibrosis, adipocyte metabolic reprogramming, and EV-mediated signaling within human adipose tissue remains limited. The platelet–adipose tissue axis should therefore be considered a working mechanistic model rather than a fully established causal pathway.

Platelet metabolic reprogramming may link systemic metabolic stress to changes in platelet effector function. Whether these metabolic alterations generate a distinct secretory phenotype capable of modifying adipose tissue biology remains to be determined.

## 5. Clinical and Translational Implications

The platelet–adipose tissue axis may link metabolic dysfunction with thromboinflammatory and cardiovascular risk. From a clinical perspective, however, well-established alterations in circulating platelet phenotype need to be distinguished from the more limited evidence directly linking these alterations to adipose tissue remodeling. This distinction is particularly relevant in obesity and type 2 diabetes, where platelet activation, adipose tissue dysfunction, and cardiovascular risk frequently coexist and may influence one another.

### 5.1. Obesity and Metabolic Dysfunction

In individuals with obesity, increased platelet reactivity is consistently observed even in the absence of overt cardiovascular disease [21,64,117]. Enhanced expression of platelet activation markers, formation of platelet–leukocyte aggregates, and elevated circulating platelet-derived EVs reflect a systemic pro-thromboinflammatory state [22,98,99,118]. These alterations occur within the same metabolic environment characterized by adipose tissue inflammation, endothelial dysfunction, insulin resistance, and oxidative stress. Platelet hyperreactivity may therefore represent not only a consequence but also an amplifier of metabolic dysfunction [16,21,24]. Through interactions with circulating immune cells and the endothelium, together with the release of soluble and vesicular mediators, activated platelets may contribute to pathways associated with adipose tissue remodeling, thereby promoting insulin resistance and chronic low-grade inflammation [10,21,77,119]. Reciprocal interaction between adipose tissue dysfunction and platelet activation may contribute to the increased cardiovascular risk associated with obesity [64,67,120].

### 5.2. Type 2 Diabetes

Type 2 diabetes further exacerbates platelet dysfunction through the combined effects of hyperglycemia, oxidative stress, and insulin resistance, resulting in impaired inhibitory signaling, altered intracellular calcium handling, enhanced responsiveness to agonists, and increased release of inflammatory mediators [22,67,68,121,122].

These abnormalities occur alongside progressive endothelial dysfunction, microvascular disease, and persistent metabolic inflammation [13,65,77,123]. Their combined effects may contribute to the accelerated vascular aging and increased thrombotic risk observed in patients with diabetes [64,124]. Diabetes may therefore provide a setting in which metabolic stress and platelet dysfunction reinforce one another. However, while platelet hyperreactivity in diabetes is well established, direct evidence that diabetic platelets accelerate adipose tissue inflammation or fibrosis in humans remains limited.

Platelet metabolic alterations may be relevant in this context. Whether specific metabolic phenotypes in diabetic platelets are associated with distinct inflammatory or vesicular profiles, and whether these profiles are associated with broader cardiometabolic dysfunction, remains unclear.

### 5.3. Cardiovascular Risk and the Metabolic–Vascular Interface

The increased cardiovascular risk associated with obesity and type 2 diabetes reflects the combined effects of dyslipidemia, hypertension, insulin resistance, chronic inflammation, endothelial dysfunction, and enhanced platelet reactivity [21,58,125]. Adipose tissue dysfunction and platelet hyperreactivity may represent interconnected components of this metabolic-vascular phenotype rather than parallel abnormalities.

Perivascular adipose tissue (PVAT) constitutes a direct anatomical interface between metabolic tissue and the vascular wall [61,62,126]. In obesity, PVAT inflammation, oxidative stress, and loss of normal vasoactive properties occur in proximity to endothelial activation and circulating platelets [126]. Platelet-derived inflammatory mediators and platelet-leukocyte interactions could contribute to local vascular inflammation, while dysfunctional PVAT may simultaneously favor platelet activation [13,61,63,127].

These reciprocal interactions may contribute to vascular remodeling and atherogenesis, providing a mechanistic bridge between adipose tissue dysfunction and cardiovascular complications [128]. This paradigm aligns with the recent clinical statement from the European Society of Cardiology, which established PVAT as an active biosensor of vascular inflammatory stress, directly linked to coronary risk stratification [129]. Nevertheless, direct demonstration of platelet-dependent signaling within human PVAT remains limited, and its contribution relative to other inflammatory and metabolic pathways has yet to be established.

### 5.4. Platelet-Based Biomarkers and Cardiometabolic Risk Stratification

Circulating markers of platelet activation, including soluble P-selectin and CD40L, platelet–leukocyte aggregates, and platelet-derived extracellular vesicles, are increased in obesity and type 2 diabetes and provide measurable indices of systemic platelet activation [13,22,61,63,98,99,127]. Their association with inflammatory and metabolic abnormalities further supports the close relationship between platelet dysfunction and cardiometabolic disease.

However, these markers are not specific to adipose tissue and cannot currently distinguish generalized platelet activation from platelet signaling directly involved in adipose tissue remodeling. This limitation is relevant to the proposed platelet–adipose tissue axis, for which no validated circulating biomarker is currently available. Platelet-derived extracellular vesicles may provide additional molecular information because their protein, lipid, and RNA cargo may reflect the state of the parent platelet, although the relationship between EV cargo and adipose tissue dysfunction remains poorly defined [130].

Integration of platelet phenotyping with metabolic and inflammatory parameters may improve the characterization of the thromboinflammatory burden in obesity and type 2 diabetes [21,22,64]. The clinical value of these markers will depend on whether they provide prognostic information beyond established cardiometabolic risk factors and whether any platelet-derived signature specific to platelet–adipose tissue interactions can be identified [131].

## 6. Experimental Models and Current Evidence

Evidence supporting interactions between platelets and adipose tissue derives from complementary in vitro, animal, and human studies. These experimental settings provide different levels of mechanistic resolution and also differ substantially in their ability to establish tissue specificity and causality. Platelet activation in obesity and type 2 diabetes is well documented, whereas direct evidence that platelets contribute to adipose tissue remodeling is more limited. The available evidence should therefore be interpreted in the context of the experimental models in which these interactions have been studied.

### 6.1. In Vitro Evidence

In vitro studies provide mechanistic evidence that platelet-derived signals can modify the function of cell populations involved in adipose tissue remodeling. Platelet releasates and individual platelet-derived mediators can enhance inflammatory signaling in monocytes and macrophages and promote endothelial activation [13,24,132,133]. Experimental studies have also identified effects of platelet-associated mediators and regulatory RNAs on pathways involved in cellular metabolism [25,26].

These systems are useful for defining individual ligand–receptor interactions and downstream signaling pathways but have important limitations. Studies based on platelet releasates or purified mediators may expose recipient cells to concentrations that do not reproduce local tissue conditions [97,98]. Moreover, isolated cell systems lack the vascular architecture, extracellular matrix, mechanical environment, and multicellular interactions characteristic of adipose tissue in vivo [5,8]. They therefore establish the biological activity of platelet-derived signals but cannot, by themselves, determine their relative contribution to adipose tissue remodeling.

### 6.2. Animal Models

Animal models provide evidence that platelet dysfunction accompanies the development of metabolic disease. Diet-induced obesity and experimental metabolic syndrome are associated with enhanced platelet activation, increased platelet–leukocyte interactions, and elevated circulating extracellular vesicles [22,99,134]. Genetic or pharmacological modulation of platelet signaling has been shown to attenuate inflammatory responses in experimental models [24,135].

Inhibition of platelet-derived mediators, including CD40L and PF4, reduces vascular inflammation and macrophage recruitment in experimental atherometabolic models, supporting a contribution of platelet-dependent pathways to these responses [13,103,135]. In addition, platelet hyperreactivity has been observed before overt vascular disease in murine models of metabolic syndrome [22,134], suggesting that platelet dysfunction can occur early during cardiometabolic disease.

Interpretation of these studies nevertheless requires caution. Most interventions targeting platelet signaling have systemic effects, making it difficult to distinguish direct actions within adipose tissue from secondary consequences of reduced vascular or systemic inflammation. This lack of tissue specificity makes adipose-specific mechanisms difficult to isolate [12,16]. Furthermore, substantial differences in platelet biology, immune composition, and adipose tissue distribution between rodents and humans limit direct translation of these findings [59,136]. Thus, current animal models provide proof of concept for platelet involvement but do not establish platelets as primary drivers of adipose tissue remodeling.

### 6.3. Human Evidence

Human studies consistently demonstrate increased platelet reactivity in obesity and type 2 diabetes, together with increased platelet–leukocyte aggregate formation and elevated circulating platelet-derived extracellular vesicles [21,63,67,98,99]. These platelet abnormalities are associated with systemic inflammation and increased cardiometabolic risk [21,64,88].

Evidence directly connecting these alterations to adipose tissue remodeling is considerably more limited. Studies combining detailed platelet phenotyping with adipose tissue sampling are scarce, and available clinical interventions have generally evaluated cardiovascular or thrombotic rather than adipose tissue endpoints [137,138]. Human adipose tissue studies have independently identified inflammatory, vascular, and fibrotic alterations associated with obesity [8,50], but the contribution of platelet-derived signals to these tissue phenotypes has not been systematically quantified. Chronic unresolved inflammation is increasingly recognized as a central component of obesity-associated adipose tissue dysfunction and cardiometabolic disease [139], further emphasizing the need to distinguish established inflammatory mechanisms from the specific contribution of platelet-derived signals.

Thus, platelet dysfunction and adipose tissue remodeling are both well documented in human metabolic disease, but direct evidence linking the two remains limited. In particular, it has not been demonstrated that platelet-derived signals reach specific adipose depots and directly modify cellular or structural remodeling in humans.

### 6.4. Current Level of Evidence and Causal Gaps

Taken together, available studies support several components of the proposed platelet–adipose tissue axis with different degrees of confidence. Platelet hyperreactivity in obesity and type 2 diabetes is well established, as are the capacity of platelets to interact with leukocytes and endothelial cells and the biological activity of several platelet-derived mediators. Experimental evidence also supports functional links between platelet-associated pathways and inflammatory responses relevant to metabolic disease.

The evidence is less direct when these mechanisms are assigned specifically to adipose tissue. The contribution of platelet-derived mediators to adipose fibrosis, the direct metabolic effects of platelet signals on adipocytes, the delivery and cellular uptake of platelet-derived extracellular vesicles within adipose depots, and the importance of depot-specific platelet interactions remain insufficiently characterized. Human evidence is particularly limited in this regard. Although extracellular vesicles are increasingly recognized as mediators of local and inter-organ communication involving adipose tissue [140], whether platelet-derived EVs cross the adipose microvascular barrier and deliver functional cargo to defined adipose cell populations remains unclear.

A further challenge is establishing directionality. Metabolic dysfunction clearly promotes platelet activation, but whether platelet alterations precede adipose tissue dysfunction, develop in parallel, or subsequently amplify established tissue remodeling remains unresolved. Current evidence therefore most strongly supports platelets as potential amplifiers of metabolic inflammation rather than as established initiators of adipose tissue remodeling.

Establishing directionality will require approaches that link platelet phenotype to tissue-level changes over time. Longitudinal studies combining platelet phenotyping with adipose tissue assessment and tissue-resolved molecular analyses could help define the temporal and spatial relationship between platelet dysfunction and adipose tissue remodeling.

## 7. Therapeutic Perspectives

The involvement of platelet-dependent pathways in metabolic inflammation raises the possibility that platelet function may represent a modifiable component of cardiometabolic disease [12,16]. However, the therapeutic implications of the platelet–adipose tissue axis remain largely unexplored. Current antiplatelet strategies are designed to prevent thrombotic events rather than to modify adipose tissue inflammation or remodeling, and direct evidence that platelet-targeted interventions improve adipose tissue outcomes is currently limited.

### 7.1. Conventional Antiplatelet Therapy

Aspirin and P2Y_12_ inhibitors are widely used to prevent atherothrombotic events in selected high-risk patients [137]. In patients with obesity, however, body mass and adiposity can influence thrombotic and bleeding risk as well as the pharmacokinetic and pharmacodynamic response to antithrombotic therapies [141]. In addition to inhibiting platelet aggregation, these agents can affect platelet degranulation, inflammatory mediator release, and platelet–leukocyte interactions [17,142]. Aspirin suppresses thromboxane-dependent platelet activation [137,143], whereas P2Y_12_ inhibition reduces platelet–leukocyte aggregate formation and can attenuate platelet-dependent inflammatory signaling [142,144]. Experimental models further demonstrate that blocking thromboxane-mediated pathways or COX-1 activity modulates vascular activation and tissue level inflammatory cell recruitment, although the direct impact on adipose remodeling remains to be fully translated to humans [145].

These effects could potentially modulate thromboinflammatory pathways associated with metabolic disease. However, clinical evidence that aspirin or P2Y_12_ inhibition directly reduces adipose tissue inflammation, fibrosis, or metabolic dysfunction is lacking. Moreover, the bleeding risk associated with systemic platelet inhibition limits the rationale for using these agents specifically to suppress metabolic inflammation. Conventional antiplatelet therapy should therefore be considered primarily a strategy for reducing thrombotic risk rather than a targeted intervention against the platelet–adipose tissue axis.

### 7.2. Modulation of Metabolic and Inflammatory Platelet Priming

Platelet hyperreactivity in obesity and type 2 diabetes is closely linked to the metabolic and inflammatory environment. Hyperglycemia, insulin resistance, oxidative stress, dyslipidemia, and chronic inflammation all contribute to abnormal platelet responsiveness [21,22,64,65,66]. Interventions that improve the underlying metabolic state may therefore indirectly normalize platelet function. In addition, some glucose-lowering therapies may influence platelet function through mechanisms extending beyond metabolic control. SGLT2 inhibitors have been associated with reduced platelet activation and oxidative stress in patients with type 2 diabetes, while experimental studies suggest direct modulatory effects on platelet responsiveness [146,147].

Weight loss interventions have been associated with improvement in several indices of platelet activation, although effects on circulating platelet-derived EVs are not uniform across studies [97,148]. These observations indicate that the platelet phenotype in metabolic disease is dynamic and at least partially reversible. They also show that platelet activation is responsive to changes in metabolic status.

Anti-inflammatory interventions provide a related example. In CANTOS, inhibition of IL-1β reduced cardiovascular events independently of lipid lowering [138], demonstrating the clinical relevance of inflammatory pathways in cardiovascular disease. Although such interventions are not platelet-directed, suppression of systemic inflammation may reduce signals that sustain platelet activation. Whether the resulting changes in platelet function contribute independently to clinical benefit, or modify platelet–adipose tissue interactions, remains unclear.

### 7.3. Targeting Platelet-Mediated Signaling

An alternative to global platelet inhibition is selective modulation of platelet-associated inflammatory pathways. CD40–CD40L signaling, PF4/CXCL4- and CCL5-dependent chemokine pathways, platelet–leukocyte interactions, and platelet-derived extracellular vesicles represent potential points of intervention [13,17,24,89].

Experimental inhibition of CD40L-dependent signaling reduces inflammatory and atherogenic responses [135,149,150], although thrombotic complications encountered with earlier anti-CD40L approaches illustrate the difficulty of targeting pathways that also contribute to normal hemostatic and immune functions [149]. Similarly, interference with platelet–leukocyte adhesion could reduce thromboinflammatory signaling without completely suppressing platelet aggregation, although its relevance to adipose tissue inflammation remains to be demonstrated.

Platelet-derived extracellular vesicles represent another potential target because they transfer proteins, lipids, and regulatory RNAs to distant cells [89]. However, therapeutic modulation of EV formation, cargo selection, or tissue delivery remains experimental, and the contribution of platelet-derived EVs to adipose tissue remodeling has not yet been sufficiently defined to support a specific therapeutic strategy.

### 7.4. Targeting Platelet Metabolic Reprogramming

The metabolic alterations of platelets in obesity and type 2 diabetes raise the possibility of targeting the intracellular processes that support the pathological platelet phenotype rather than globally inhibiting platelet activation [16,77,78]. Changes in glycolytic activity, mitochondrial function, redox balance, and calcium signaling may influence platelet responsiveness and effector functions [79]. Whether these metabolic alterations also determine the composition of platelet releasates or extracellular vesicle cargo remains insufficiently defined.

In principle, metabolic modulation might alter pathological platelet signaling while preserving essential hemostatic functions. However, platelet bioenergetic pathways are closely integrated with normal platelet activation, and the metabolic features that specifically distinguish pathological from physiological platelet responses remain incompletely defined. Moreover, it is not yet established whether metabolic reprogramming produces a disease-specific secretory phenotype that can be selectively targeted.

A better definition of the relationship between platelet metabolism and effector output is therefore needed before these pathways can be considered therapeutic targets. Identification of metabolic pathways that preferentially regulate inflammatory or vesicular signaling, without substantially impairing hemostasis, could provide a more selective approach to platelet modulation in cardiometabolic disease. At present, however, this possibility remains primarily a mechanistic and preclinical concept.

**Figure 4:**
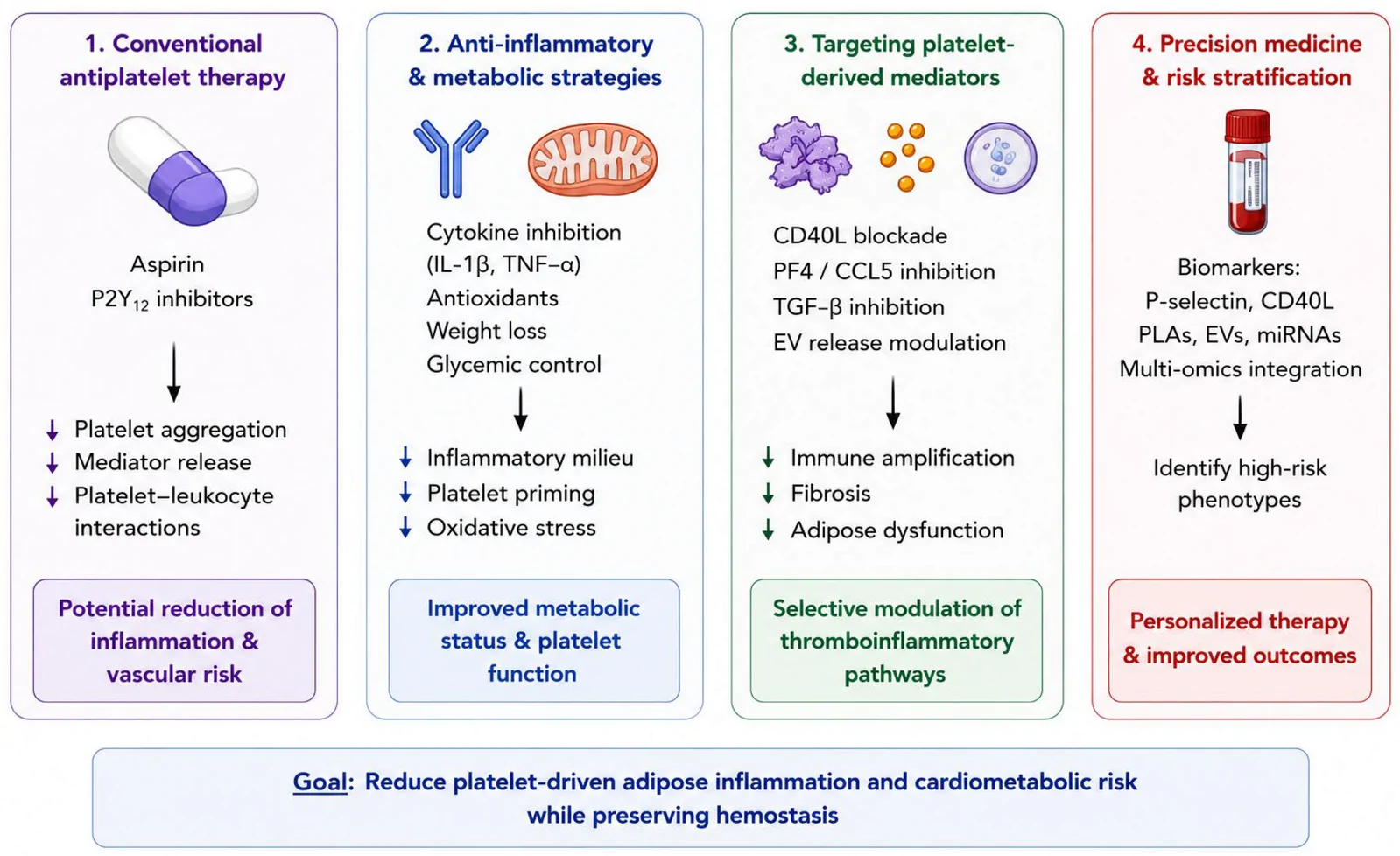
Therapeutic Opportunities. Potential clinical interventions are stratified into four strategic axes: (1) conventional antiplatelet therapies (aspirin, P2Y_12_ inhibitors) to reduce aggregation and thromboinflammatory signaling; (2) metabolic and systemic anti-inflammatory strategies (cytokine inhibition, antioxidants, weight loss, glycemic control) to lower oxidative stress and platelet priming; (3) targeting platelet-associated inflammatory signaling pathways (CD40L blockade, PF4/CCL5/TGF-β inhibition); and (4) precision medicine and cardiovascular risk stratification utilizing circulating platelet activation markers, extracellular vesicles, and integrated multi-omics. CD40L, CD40 Ligand; EVs, Extracellular Vesicles; IL-1β, Interleukin-1 beta; miRNAs, MicroRNAs; PF4, Platelet Factor 4; PLAs, Platelet-Leukocyte Aggregates; TNF-α, Tumor Necrosis Factor alpha. The figure was generated using ChatGPT-5.6 sol Enterprise (OpenAI; DALL·E).

Taken together, current and emerging therapeutic strategies may act at different levels of the platelet–adipose tissue axis, ranging from conventional inhibition of platelet activation to modulation of metabolic priming and platelet-derived inflammatory signaling (Figure 4).

## 8. Knowledge Gaps and Future Directions

The platelet–adipose tissue axis integrates several established features of metabolic disease, including platelet hyperreactivity, endothelial dysfunction, chronic inflammation, and maladaptive adipose tissue remodeling. What remains uncertain is the extent to which these processes are causally connected. In particular, it is not yet clear whether metabolic alterations generate a distinct platelet phenotype that directly modifies adipose tissue biology, or whether platelet dysfunction predominantly reflects the systemic inflammatory and metabolic environment. Addressing this question requires moving beyond parallel measurements of platelet and adipose tissue dysfunction to approaches that resolve their temporal, spatial, and molecular relationships.

### 8.1. Establishing Temporality and Tissue Specificity

The temporal relationship between platelet dysfunction and adipose tissue remodeling is not clear. Platelet hyperreactivity can occur early in metabolic disease [21,22,134], but whether it precedes the development of adipose inflammation and fibrosis or emerges in parallel with metabolic and endothelial dysfunction remains unclear. Longitudinal studies combining platelet phenotyping with repeated metabolic and adipose tissue assessments could establish whether specific platelet alterations precede, accompany, or follow the transition from adaptive adipose expansion to tissue dysfunction.

Establishing causality also requires greater tissue specificity. Systemic modulation of platelet function cannot readily distinguish direct platelet effects within adipose tissue from secondary changes caused by reduced vascular or systemic inflammation. Experimental approaches that selectively manipulate platelet-derived signals, together with direct assessment of adipose tissue responses, are therefore needed to determine the contribution of platelets independently of other cellular sources of the same mediators.

### 8.2. Does Metabolic Reprogramming Alter the Platelet Secretory Phenotype?

A central question raised by the platelet–adipose tissue model is whether metabolic alterations change the functional output of platelets beyond quantitative changes in activation. Obesity and type 2 diabetes alter platelet bioenergetics, mitochondrial function, redox balance, and intracellular signaling [16,19,21,77,79], but the relationship between these metabolic changes and the composition of platelet-derived signals remains poorly defined. Recent evidence from metabolic liver disease provides proof of principle that alterations in platelet mitochondrial phenotype can be reflected in platelet-derived EVs and can influence metabolic responses in recipient cells [151]. However, whether analogous mechanisms operate in obesity-associated adipose tissue remains unknown.

An important question is whether changes in glycolytic and mitochondrial metabolism influence granule secretion and extracellular vesicle formation, and whether the metabolic state of platelets also contributes to cargo selection [152]. In parallel, experimental studies indicate that platelet-derived EVs can be functionally tuned by the mode of platelet activation, with receptor-specific stimulation generating EV populations with distinct immunomodulatory properties and differences in protein and miRNA profiles [133]. Whether metabolic reprogramming similarly influences EV composition or function remains unknown.

Integrated analysis of platelet metabolism with proteomic, lipidomic, and RNA profiling of platelet releasates and extracellular vesicles could determine whether metabolically altered platelets acquire a reproducible inflammatory, profibrotic, or metabolism-modulating secretory phenotype [116].

If metabolic reprogramming alters only the magnitude of conventional platelet activation, it may primarily reflect enhanced platelet reactivity. In contrast, identification of a reproducible disease-associated secretory phenotype would provide evidence that metabolic disease modifies platelet effector function beyond hyperreactivity.

### 8.3. Resolving Cell- and Depot-Specific Communication

The cellular targets of platelet-derived signals within adipose tissue remain incompletely characterized. Macrophages and endothelial cells are supported by the strongest mechanistic evidence, whereas direct effects on adipocytes, fibroblasts, and stromal progenitor populations are less well defined. Similarly, the relative contributions of direct platelet–cell interactions, soluble mediators, and extracellular vesicles are likely to depend on both cell type and local microenvironment.

Several pathways discussed in this review illustrate these uncertainties. PF4/CXCL4 can influence monocyte and macrophage phenotypes [24,104], but its platelet-specific contribution to macrophage reprogramming within obese adipose tissue remains unclear. Peripheral serotonin regulates adipocyte lipid metabolism and thermogenic pathways [23,115], yet the contribution of platelet-derived serotonin to these effects has not been established. Likewise, platelet-derived extracellular vesicles carry regulatory miRNAs, including miR-223 [25,26], but their delivery to specific adipose cell populations and their functional effects in vivo remain poorly characterized. By contrast, the role of adipose tissue-derived EVs in local and inter-organ metabolic communication is increasingly well characterized [140], highlighting the need to determine whether a reciprocal platelet-to-adipose EV signaling axis operates in vivo.

These interactions may also differ substantially among adipose depots. Visceral, subcutaneous, and perivascular adipose tissues differ in vascular density, endothelial phenotype, immune composition, and inflammatory activity [58,59,60,62]. Mapping platelet-derived signals and their cellular targets across these depots could identify where platelet–adipose communication is most relevant in vivo.

### 8.4. Translating the Platelet–Adipose Axis to Human Disease

The major translational gap is the lack of human studies that simultaneously characterize platelet phenotype and adipose tissue biology. Most clinical studies measure circulating platelet activation, whereas adipose tissue studies independently characterize inflammation, fibrosis, or metabolic dysfunction. Studies directly integrating these two compartments remain uncommon.

Studies combining platelet functional and metabolic profiling with adipose tissue biopsies could directly test whether specific platelet phenotypes are associated with defined tissue-level changes. Spatial and single-cell approaches may be particularly informative for identifying platelet-associated signals within vascular and perivascular niches and for resolving the cellular populations exposed to these signals [153,154,155,156]. Parallel profiling of platelet-derived extracellular vesicles could determine whether circulating EV signatures are associated with molecular changes within adipose tissue.

Current platelet biomarkers, including soluble P-selectin, CD40L, platelet–leukocyte aggregates, and platelet-derived extracellular vesicles, primarily measure systemic platelet activation and do not identify platelet–adipose tissue communication specifically [17,98]. A more informative biomarker would require a molecular signature that links a defined platelet phenotype to a corresponding tissue response. Such a relationship would provide mechanistic support for the platelet–adipose tissue connection and could help establish its clinical relevance in cardiometabolic disease.

## 9. Conclusions

Obesity-associated adipose tissue remodeling results from the interplay of metabolic stress, chronic inflammation, extracellular matrix remodeling, and vascular dysfunction. In parallel, obesity and type 2 diabetes are characterized by platelet hyperreactivity and alterations in platelet metabolism, redox balance, secretory activity, and extracellular vesicle release. Together, these changes provide the biological basis for considering platelets as potential contributors to the link between metabolic and vascular dysfunction.

Platelet-derived signals can regulate leukocyte recruitment, endothelial activation, macrophage phenotype, fibroblast function, and cellular metabolism through direct interactions, soluble mediators, and extracellular vesicles. These observations support a model in which metabolically altered platelets may act as amplifiers of inflammatory, vascular, and structural pathways relevant to adipose tissue dysfunction, while the extent of direct platelet-dependent remodeling within adipose tissue remains uncertain. However, the strength of evidence differs across these mechanisms, and direct demonstration of platelet-dependent adipose tissue remodeling, particularly in humans, remains limited.

A key unresolved question is therefore whether metabolic disease simply increases platelet reactivity or qualitatively modifies platelet effector function. Whether such alterations generate a reproducible secretory phenotype capable of influencing adipose tissue biology remains an open question. Resolving this issue will help determine whether platelet metabolic reprogramming is primarily a marker of systemic metabolic stress or a modifiable contributor to cardiometabolic disease.

## Figures and Tables

**Table 1:** Molecular mechanisms contributing to platelet hyperreactivity and metabolic reprogramming in obesity and T2D.

Metabolic Context/Stimulus	Molecular Mechanism	Platelet Consequence	Evidence/Model	Ref.
Hyperglycemia	Mitochondrial superoxide production → enhanced Syk phosphorylation during collagen signaling	Increased platelet responsiveness	Human platelets; ex vivo/in vitro hyperglycemia	[70]
Diabetes/hyperglycemia	Altered Na^+^/Ca^2+^ exchange and Ca^2+^ homeostasis	Enhanced intracellular Ca^2+^ responses	Human diabetic platelets; in vitro high-glucose exposure	[71]
Obesity/T2D; insulin resistance	Impaired insulin-mediated inhibitory signaling	Reduced antiaggregatory effect of insulin	Human platelets; obesity and T2D	[74]
Dyslipidemia/oxLDL	CD36 → Src/PKC → NOX2-derived ROS; impaired cGMP/PKG signaling	Enhanced platelet activation	Human platelets; mechanistic ex vivo/in vitro study	[75]
T2D/oxLDL	Increased platelet CD36 N-glycosylation → Lyn–JNK signaling	Increased responsiveness to oxLDL	Human platelets; patients with T2D	[76]
Platelet activation	Increased glucose consumption and redistribution of central carbon metabolism	Metabolic adaptation supporting platelet activation	Human platelets; ^13^C metabolic flux analysis	[80]
T2D with vascular complications	Impaired mitochondrial respiration, altered NAD^+^/NADH balance, increased glycolytic flux and mitochondrial oxidant generation	Altered platelet bioenergetic phenotype	Human platelets; patients with T2D	[83]
Megakaryocyte differentiation	Remodeling of fatty-acid uptake and de novo lipid synthesis	Megakaryocyte maturation and proplatelet formation	Experimental megakaryocyte models	[85]
Diet-induced obesity	Adipocyte-derived fatty-acid transfer to megakaryocytes via CD36-dependent mechanisms	Enhanced megakaryocyte maturation/proplatelet formation	Mouse model of HFD-induced obesity	[86]

**Table 2:** Candidate molecular mediators of the platelet–adipose tissue axis and their adipose-relevant cellular targets.

Mediator/Cargo1	Platelet Source	Target in Adipose Tissue	Reported/Potential Biological Effect	Refs.
CD40L	α-Granules	Endothelial Cells/Monocytes	Can activate inflammatory signaling in endothelial cells/monocytes; adipose-specific ef-fects remain to be established	[103]
PF4/CXCL4	α-Granules	Monocytes/Macrophages	Promotes monocyte survival and differentiation toward macrophages; its platelet-specific contribution to macrophage reprogramming within adipose tissue remains unclear	[104]
TGF-β1	α-Granules	Fibroblasts/stromal cells	Platelets constitute a res-ervoir of TGF-β1 with es-tablished profibrotic activ-ity; the contribution of platelet-derived TGF-β1 to adipose tissue fibrosis re-mains unestablished	[27,106]
PDGF	α-Granules	Fibroblasts/adipose stromal progenitors	Platelet PDGF promotes fibroblast proliferation and chemotaxis, while PDGFRα signaling can drive profibrotic conversion of adipose progenitors; a direct platelet-PDGF–adipose fibrosis pathway has not been established	[107,108,110]
Serotonin (5-HT)	Dense Granules	Adipocytes	Peripheral serotonergic signaling regulates lipid metabolism and thermogenic programs; whether platelet storage and release materially contribute to these adipose effects remains unestablished	[23,115]
Platelet sEVs (miR-223)	Platelet membrane/EV cargo	Endothelial cells; potentially other adipose-relevant cell populations	Platelet EVs can transfer functional miRNA cargo to endothelial cells and modify recipient-cell gene expression; delivery to and functional effects in adipose-resident cells remain to be established	[25]

## Data Availability

No new data were created or analyzed in this study. Data sharing is not applicable to this article.

## Abbreviations

The following abbreviations are used in this manuscript:

5-HT	5-Hydroxytryptamine (Serotonin)
5-HT2A	5-Hydroxytryptamine (Serotonin) Receptor 2A
5-HT2B	5-Hydroxytryptamine (Serotonin) Receptor 2B
BDNF	Brain-Derived Neurotrophic Factor
CCL2	C-C Motif Chemokine Ligand 2
CCL5	C-C Motif Chemokine Ligand 5
CD40	Cluster of differentiation 40
CD40L	CD40 Ligand
CXCL4	C-X-C Motif Chemokine Ligand 4
DAMPs	Damage-Associated Molecular Patterns
ECM	Extracellular Matrix
EVs	Extracellular Vesicles
HIF-1α	Hypoxia-Inducible Factor 1 Alpha
ICAM-1	Intercellular Adhesion Molecule 1
IL-1β	Interleukin-1 Beta
IL-1R	Interleukin-1 Receptor
IL-6	Interleukin-6
miR-126	MicroRNA-126
miR-223	MicroRNA-223
miRNAs	MicroRNAs
MCP-1	Monocyte Chemoattractant Protein-1
MMe	Metabolically Activated Macrophages
NADPH	Nicotinamide Adenine Dinucleotide Phosphate
NF-κB	Nuclear Factor Kappa B
NOX	NADPH Oxidase
P2Y12	Purinergic receptor P2Y, G-protein coupled 12
PDGF	Platelet-Derived Growth Factor
PF4	Platelet Factor 4
PLAs	Platelet–Leukocyte Aggregates
PVAT	Perivascular Adipose Tissue
PSGL-1	P-Selectin Glycoprotein Ligand-1
ROS	Reactive Oxygen Species
sEVs	Small Extracellular Vesicles
SGLT2	Sodium–Glucose Cotransporter 2
T2D	Type 2 Diabetes
TGF-β	Transforming Growth Factor Beta
TNF-α	Tumor Necrosis Factor Alpha
